# Neurobiological basis for the application of yoga in drug addiction

**DOI:** 10.3389/fpsyt.2024.1373866

**Published:** 2024-04-18

**Authors:** Nilkamal Singh

**Affiliations:** Department of Yoga, Manipur University, Manipur, India

**Keywords:** yoga, addiction, neurobiology, reward, stress, inhibitory control

## Introduction

Studies on neurobiology of addiction suggests that addiction is a three-stage cycle consisting of (i) binge/intoxication (ii) withdrawal/negative effect, and (iii) preoccupation/anticipation. Prolonged drug use can increase the severity of this cycle and dysregulate the brain reward system leading to (i) increased substance seeking when exposed to substance related cues (ii) decrease the sensitivity of brain reward system; increase the sensitivity of brain stress system, and (iii) decrease in executive function (1). In the initial stage of addiction, impairments can be seen in the brain reward circuitry and it gradually expands to higher order processes which controls emotions, cognition and behavior (2). This results in reduced sensitivity to motivation towards actions which are not related to drugs, increased sensitivity of the emotional circuits to stress and impaired capacity to self-regulate (3). These changes in the brain can continue for months or years after abstinence from drugs and can raise difficulties in the rehabilitation process of drug addicts. Hence, management of addiction should address the dysregulation of the brain reward circuitry, stress mechanism and inhibitory control for positive outcome.

Among non-pharmacological interventions having positive impact on the brain, yoga is emerging as an effective intervention. Yoga is a mind-body practice which involves following certain ethical principles (*Yamas* and *Niyamas*), practicing physical postures (*asanas*), cleansing practices (*kriyas*), breath regulation (*pranayama*), sense control (*pratyahara*), concentration (*dharana*) and meditation (*dhyana*). Since the practice of yoga involves postural control, breath regulation, maintenance of interoceptive awareness, regulation of emotion and attentional control, it is postulated that both top-down and bottom-up mechanism of interaction between the brain and peripheral tissues are involved during yoga practice. Through these mechanisms yoga can bring changes in the psychophysiology of the practitioners. Although there is scarcity of scientific literature on the effect of yoga and the neurobiology of addiction, several studies have reported the beneficial impact of yoga on brain reward system, stress management and inhibitory control.

## Yoga and brain reward system

It is well documented that dopamine plays an important role in the reward circuitry as it regulates the reward value of food, drink, sex, social interaction, and substance abuse (4). In drug addiction, the rewarding effect of addictive drugs is produced by interfering with the brain’s dopamine system (5). Evidence suggests that drugs of abuse can cause transient increase in dopamine level which can result in the activation of dopamine D1 receptors leading to subjective experience of euphoria during intoxication (6). If left unchecked, drug abuse can cause reward deficits due to decrease in dopamine neuronal firing and increased stress (7). The D1 receptor is also linked with conditioning and memory mechanisms that can intensify the reinforcing effects of drugs of abuse (8). Hence, an increase in the expression of D1 receptors can play an important role in the onset and maintenance of addiction. Another important change brought by drug addiction in the dopamine system is the decreased expression of dopamine D2 receptors (9–12). Decrease in D2 receptors is associated with reduced activity of the orbito-frontal cortex, anterior cingulated gyrus and dorsolateral prefrontal cortex in drug addicts. These regions of the brain are associated with executive functions and inhibitory control (13). Hence, a decrease in D2 receptors can contribute in increasing compulsive behavior in drug addicts.

A recent study revealed that Rajyoga meditation can bring grey matter volume changes in regions of the brain that regulates reward and happiness (14). This study compared the grey matter volume in reward processing areas of the brain between Rajyoga meditation practitioners and non-meditators. Grey matter volume in the right superior frontal gyrus and left inferior orbitofrontal cortex and bilateral precuneus was found to be higher in the Rajyoga meditation practitioners. Neuroimaging studies on drug addicts reported grey matter volume changes in these regions of the brain. A study reported positive correlation between grey matter volume in the superior frontal gyrus and duration of abstinence (15). The same study also found a positive correlation with grey matter volume in the right precuneus with duration of abstinence and a negative correlation was observed with years of substance used. A separate study reported reduced grey matter volume in the orbitofrontal cortex of cocaine addicts (16). Hence, Rajayoga meditation can improve grey matter volume changes in the regions of the brain affected by drug addiction. Apart from these structural changes, meditation is also reported to increase endogenous dopamine release (17). During yoga nidra meditation there was a 7.9% reduction in ^11^C-raclopride binding in the ventral striatum indication a 65% increase in endogenous dopamine release. An increased striatal dopamine binding to D2 receptors was also observed in this study. The ventral striatum is associated with the acquisition and development of reward-based behaviors and has implications in drug addiction and drug-seeking behaviors (18). The increased dopamine binding to D2 receptors may also have implications in the management compulsive behavior and executive functions in drug addicts.

## Yoga and neurobiology of stress

In the withdrawal/negative affect stage of addiction, stress functions as an important source of motivation for compulsive drug seeking and contributes largely to the transition from drug abuse to addiction stage. The withdrawal of drug act as a stressor leading to the activation of brain stress system. A growing body of evidence suggests that yoga can be effective in the reduction of stress. Studies on yoga and autonomic nervous system activity reported a shift towards vagal dominance following yoga practice indicating an increase in parasympathetic activity. This is supported by findings from the studies on yoga and heart rate variability. A study conducted on medical students assessed the effect of pranayama on heart rate variability, anxiety, memory and psychological well-being (19). The study reported that after practicing pranayama for 6 months there was a reduction in the low frequency component and an increase in the high frequency component of heart rate variability, indicating an increase in parasympathetic activity. There was also an improvement in the anxiety scores and psychological well-being of the students. A study on the effect of Zen meditation and hear rate variability of drug abusers reported an increase in time domain components of heart rate variability following the practice of Zen meditation (20). This showed that Zen meditation can increase parasympathetic activity in drug abusers. Apart from this effect on the heart rate variability yoga is also reported to reduce plasma cortisol level. A study evaluated the effect of Sudarshan Kriya yoga on the plasma cortisol and adenocorticotropic hormone levels of inpatients of alcohol dependence. The study concluded that practicing Sudarshan Kriya Yoga for two weeks can lower stress-hormone levels (plasma cortisol and adenocorticotropic hormone) in patients with alcohol dependence (21). One of the most plausible mechanism proposed for these changes in the autonomic nervous system and stress hormone following the practice of yoga is through the stimulation of respiratory vagus nerve. In 2018, Gerritsen and Band suggested a two-way model (direct and indirect) for the respiratory stimulation of the vagus nerve (22). In the direct pathway, slow and longer exhalation which is important for yoga practice (*asana*, *pranayama* and *dhyana*) is directly linked with the vagal nerve as it controls slowing of respiration and exhalation. The indirect pathway follows the physiological feedback theory where slow and restful breathing generates a physiological body pattern associated with relaxation and low threat situation. This information is projected to the central nervous system through the vagal afferents leading to the reinforcement of the rest and digest state of the body through its top-down mechanism.

## Yoga and inhibitory control

In chronic drug addicts a compulsive pattern of drug seeking and uncontrolled intake is observed. This compulsive behavior is attributed to the dysregulation of the brain’s inhibitory mechanism because of prolonged drug use. In addicted individuals, dysregulation of the anterior cingulate, dorsolateral prefrontal cortex and orbitofrontal cortices is reported (2). These regions of the brain are responsible for inhibitory control over reward related behavior. The practice of yoga requires maintenance of awareness about the object of attention, respiratory sensations and interoceptive feedback from body sensations and mental activity. Such interoceptive awareness helps in the inhibition of emotional and behavioral distractions (23). Studies have implicated yoga in improving emotional and cognitive control. A study compared the interference of emotional stimuli on executive task performance between yoga practitioners and controls with no experience in yoga using functional magnetic resonance imaging. The study reported that the prefrontal activation during negative emotional stimuli in the yoga practitioners was higher than the control group. This change in the prefrontal cortex was observed only while performing a cognitively demanding task suggesting that yoga practitioners were able to selectively recruit frontal executive mechanism to counter emotional distractions (24). Another study using functional near infrared spectroscopy reported an increase in oxyhemoglobin level in the prefrontal cortex during Flanker task after practicing yoga meditation (YoMed) for 15 minutes daily for 5 days (25). The study concluded that YoMed was effective in increasing inhibitory control in young adults. Increase in inhibitory control following yoga practice was also reported in a study on smokers with nicotine dependence (26). To assess inhibition, the participants performed a Go/Nogo task consisting of Smoking-Go, Smoking-Nogo, Neutral-Go, and Neutral-Nogo stimulus conditions. Event related potential (ERP) N2 and P3 amplitudes and latencies were also recorded. This study observed that a single session of yoga can increase inhibitory control in smokers with nicotine dependence. A separate study on addicted population showed improved self-control ability in emotion regulation and increased anterior cingulate cortex (ACC) and prefrontal cortex (mPFC) activity after meditation (27). Apart from this, repeated practice of attentional and emotional regulations is associated with structural changes in the brain. A MRI study on 20 meditators reported higher cortical thickness in the prefrontal cortex of the meditators compared to control group (28). Hence yoga can help in improving inhibitory control in healthy and addicted individuals indicating a possible application of yoga in prevention and management of drug addiction.

## Conclusion

Drug addiction is associated with neurobiological changes in the brain leading to dysregulation of the brain’s reward system, stress system and inhibitory mechanism. This can bring behavioral changes characterized by compulsive drug seeking and uncontrolled drug intake. The process of rehabilitation of drug addicts will be efficient if these dysregulations can be countered. Among non-pharmacological measures to manage drug addiction, yoga can be a useful intervention. Yoga has been shown to improve the brain’s reward system by bringing morphological and dopaminergic changes in the regions of the brain associated with reward circuitry. Since study on yoga and reward system of drug addicts is not available, it is not possible to conclusively claim the benefits of yoga in the reward system of drug addicts. However, findings of the studies conducted on healthy participants showed encouraging results about the effect of yoga on the brain’s reward system and can be the basis of future studies on yoga and drug addicts. Studies on the effect of yoga on stress system showed that yoga can reduce stress by improving the neurobiological determinants of stress. Few studies have shown that yoga can bring these changes in drug addicts also. Similarly, there are evidences about the effectiveness of yoga in improving inhibitory control of drug addicts by bringing functional and structural changes in the brain. Since yoga has been shown to improve brain’s reward system, stress system and inhibitory control, it can be speculated that yoga can be useful in the management of drug addiction.

## Author contributions

NS: Conceptualization, Writing – original draft, Writing – review & editing.
